# Distribution of substance P and the calcitonin gene-related peptide in the human tensor tympani muscle

**DOI:** 10.1007/s00405-013-2469-1

**Published:** 2013-04-09

**Authors:** Masahiko Yamazaki, Iwao Sato

**Affiliations:** Department of Anatomy, School of Life Dentistry at Tokyo, Nippon Dental University, 1-9-20 Fujimi, Chiyoda-ku, Tokyo 102 -8159 Japan

**Keywords:** Tensor tympani muscle, Calcitonin gene-related peptide, Substance P, Immunohistochemistry

## Abstract

Substance P-immunoreactive nerve fiber (SP-IR NF) and calcitonin gene-related peptide-immunoreactive nerve fiber (CGRP-IR NF) are important mediators of neurogenic inflammation and blood supply. SP-IR and CGRP-IR NFs in the tensor tympani muscle (TTM) of the human middle ear have yet to be described. In this study, the TTM, tympanic membrane, malleus in the middle ear and tensor veli palatini muscle (TVPM) were examined by whole-mount immunohistochemistry in tissue from Japanese subjects. Thirteen human cadavers (ranging in age from 46 to 90 years) were used in this study. SP-IR and CGRP-IR NFs were primarily found on vessels at the origin, insertion and belly of the surface of the TTM and on the internal surface of the tympanic membrane. These neural factors were also detected on the surface of the malleus and the insertion of the TVPM. Therefore, our results indicate that existence of the SP-IR and CGRP-IR NFs of the TTM and the TVPM may reflect muscle properties involved in pain or inflammation of the middle ear.

## Introduction

Substance P-immunoreactive nerve fiber (SP-IR NF) and calcitonin gene-related peptide-immunoreactive nerve fiber (CGRP-IR NF) have been found in humans and other species in connective tissue beneath epithelium, around blood vessels, in the nasal mucosa and in the mucous glands in the respiratory tract [1–7]. SP-IR and CGRP-IR NFs are also present in the mucosa in obstructive sleep apnea patients [8]. In addition, perivascular distribution of SP-IR and CGRP-IR NFs on the surface of skeletal muscle has been reported [9–11]. The presence of SP-IR and CGRP-IR NFs indicates vascular regulation, such as vasodilation or vasoconstriction [12]. SP-IR and CGRP-IR NFs have been found in mammalian middle ear mucosa [13, 14]. However, the distribution of SP-IR and CGRP-IR NFs in the nerve roots and blood vessels of the human TTM has not previously been reported. Moreover, the presence and distribution of CGRP-IR NFs indicate sensory transmission and the regulation of local blood flow, smooth muscle tone and glandular secretion in the upper and lower respiratory tract of several mammals, including humans [1]. Previous studies have examined local regional sites; we aimed to study distribution of neurovascular insertion sites such as sensory nerve fiber [4], which indicate morphological and functional properties of middle ear muscles. Therefore, the present study used whole-mount immunohistochemical analysis to examine the distribution of SP-IR and CGRP-IR NFs in the human middle ear, particularly in the TTM.

## Subjects and methods

**Fig. 1:**
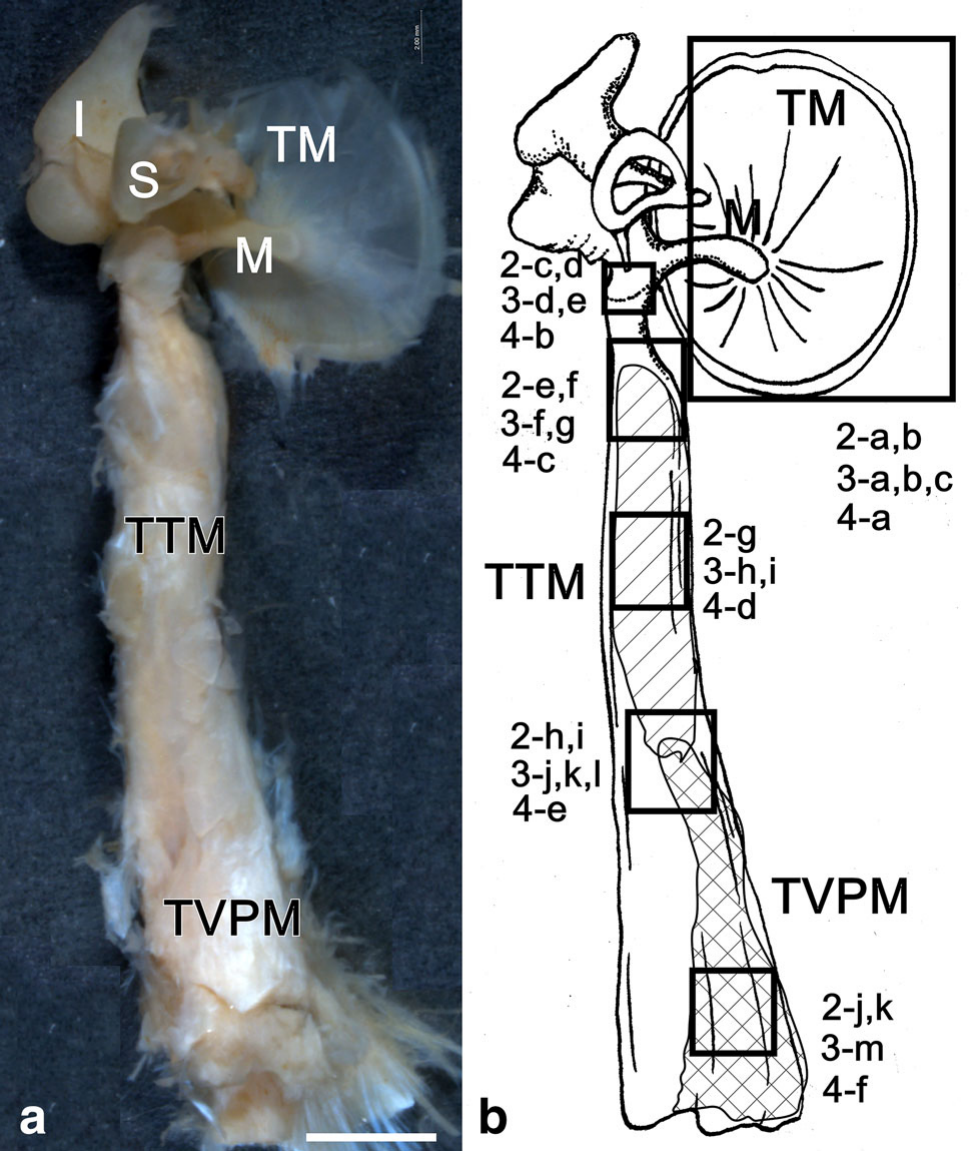
Inner side views of the human right *TTM* ear ossicles (*M* malleus, *I* incus, and *S* stapes), and TM (60-year-old male) (**a**) The observed sites are shown in **b** 2-*a*, *b*. 3-*a*-*c*, 4-*a* inner surface of the tympanic membrane; 2-*c*, *d*, 3-*d*, *e*, 4-*b* neck of the malleus; 2-*e*, *f*, 3-*f*, *g*, 4-*c* insertion of the TTM at the malleus; 2-*g*, 3-*h*, *I*, 4-*d* belly of the TTM, 2-*h*, *i*, 3-*j*-*l*, 4-*e* connection region between the TTM and TVPM; 2-*j*, *k*, 3-*m*, 4-*f* belly of the TVPM; *bar* 2 mm

In this study, the middle ears of 13 human cadavers aged 46–90 years (mean 78.5 ± 13.3 years old; male, *n* = 5, 70.8 ± 15.6; female, *n* = 8, 80.5 ± 12.6 years old) were examined. All human cadavers had been donated for dissection. Samples were injected with 10 % formalin with return perfusion via the femoral artery. After anatomical dissection, the middle ear (including the TTM and the TVPM) was removed from the temporal bone (see Fig. 1). We examined the following six surface sites: the inner surface of the tympanic membrane (TM), neck of the malleus, insertion of the TTM at the malleus, belly of the TTM, connection region between the TTM and TVPM, and belly of the TVPM (Fig. 1a, b). The samples were then used for immunohistochemical analysis of the middle ear muscles and ossicles.

## Whole-mount immunohistochemistry

Whole-mount specimens were washed with distilled water for 24 h, incubated with 3 % H_2_O_2_ for 20 min to eliminate endogenous peroxidase activity, and digested with 0.02 % proteinase K (Wako, Tokyo, Japan) for 1 h at 38 °C. After overnight fixation in 4 % paraformaldehyde, the samples were washed with distilled water for 50 min, and the proteinase K digestion and overnight fixation steps were repeated. The samples were then washed with phosphate-buffered saline (PBS) for 30 min and sequentially incubated in 2.5, 5 and 10 % sucrose in PBS (1 h for each stage). After overnight incubation at 4 °C in PBS containing 2 % Triton X-100, the samples were washed with PBS (1 h for each wash) and incubated for 1 h at room temperature with 2 % normal goat serum/PBS (pH 7.2) containing 0.05 % Tween 20 to prevent non-specific antibody binding. Right middle ear samples were then incubated for 2 days at 4 °C with rabbit polyclonal antibodies against SP (diluted 1:100; Lab Vision, CA, USA) and CGRP (1:1,000; Biogenesis, NH, USA) or with normal goat serum as the negative control (left middle ears). The samples were then washed three times with PBS for 1 h each wash. Whole-mount samples were incubated with horseradish peroxidase-conjugated goat anti-rabbit IgG (Santa Cruz Biotechnology, USA) following the manufacturer’s instructions. The samples were then washed with PBS three times for 1 h. The staining was visualized using 0.02 % H_2_O_2_ and 0.1 % (1 mg/ml) diaminobenzidine tetrahydrochloride in 0.1 M Tris–HCl, pH 7.2. Appropriate negative controls were included. Images were acquired using a stereomicroscope (Leica MZ 16FA; Leica Microsystems, USA) with the Leica Application Suite software (Leica Microsystems).

## Ethics

Cadavers were obtained by consensual donation, according to guidelines from the law concerning body donation and the law concerning cadaver dissection and preservation.

## Results

### Distribution of SP-IR and CGRP-IR NFs

**Fig. 2:**
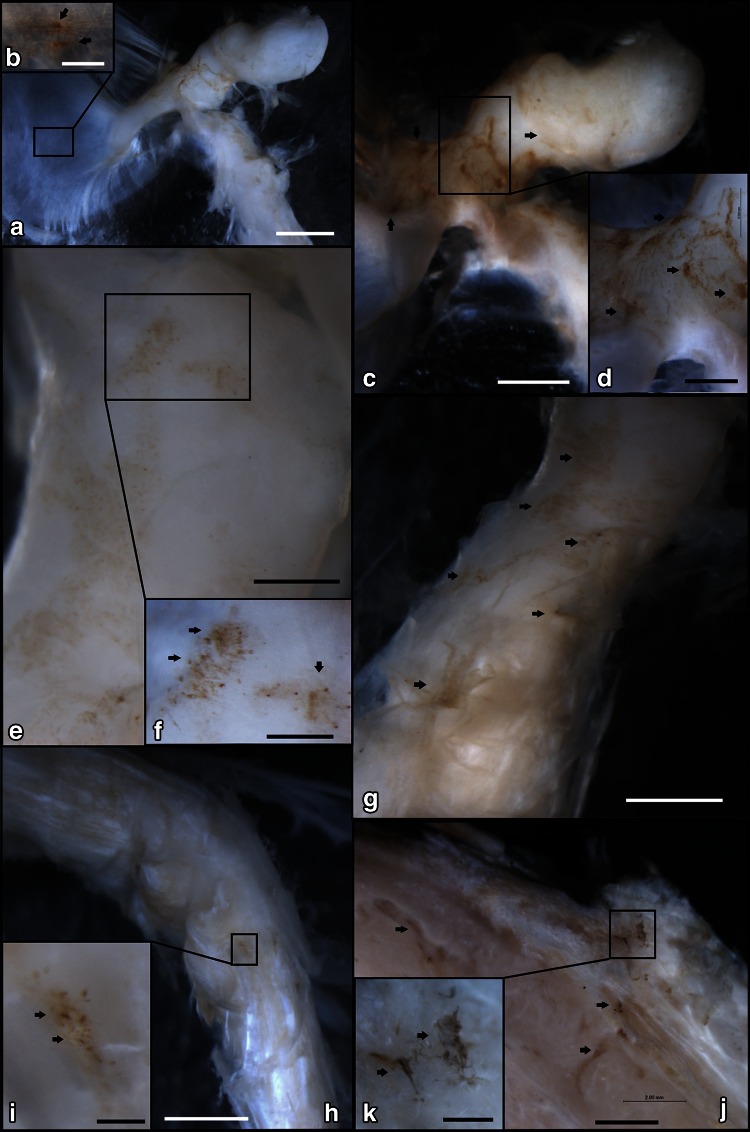
The distribution of Substance P-immunoreactive nerve fibers (SP-IR NFs) in the inner surface region of the middle ear shown by immunohistochemical staining at the macroscopic level. **a** SP-IR NFs were found in the inner surface of the TM and malleus by anti-Substance P immunohistochemical reactions (76-year-old, female) (*bar* 2 mm). **b** Higher magnification of the *square* in **a**. SP-IR NFs (*arrows*) were sparsely distributed on the TM (*bar* 0.25 mm). **c** Higher magnification of neck of the malleus (see Fig. **a**) (*bar* 1 mm). **d** Higher magnification of the *square* in **c**. Numerous SP-IR NFs (*arrows*) were clearly observed (bar = 0.5 mm). **e** The insertion site of the TTM on the malleus (see Fig. 1) (*bar* 0.5 mm); **f** Higher magnification of the *square* in **e**. Mesh-like structures of Substance P immunoreactivity (*arrows*) were clearly observed (*bar* 0.25 mm). **g** The belly of the TTM (see Fig. 1). A few SP-IR NFs (*arrows*) were sparsely distributed on the TTM (*bar* 1 mm). **h** The connection site between the TTM and the TVPM (see Fig. 1) (*bar* 2 mm); **i** higher magnification of the *square* in **h**. A mesh-like structure of Substance P immunoreactivity (*arrows*) was observed (*bar* 0.25 mm). **j** The belly of the TVPM (see Fig. 1) (*bar* 2 mm); **k** Higher magnification of the *square* in **j**. A mesh-like structure or fibers of Substance P immunoreactivity (*arrows*) was observed (*bar* 0.5 mm)

**Fig. 3:**
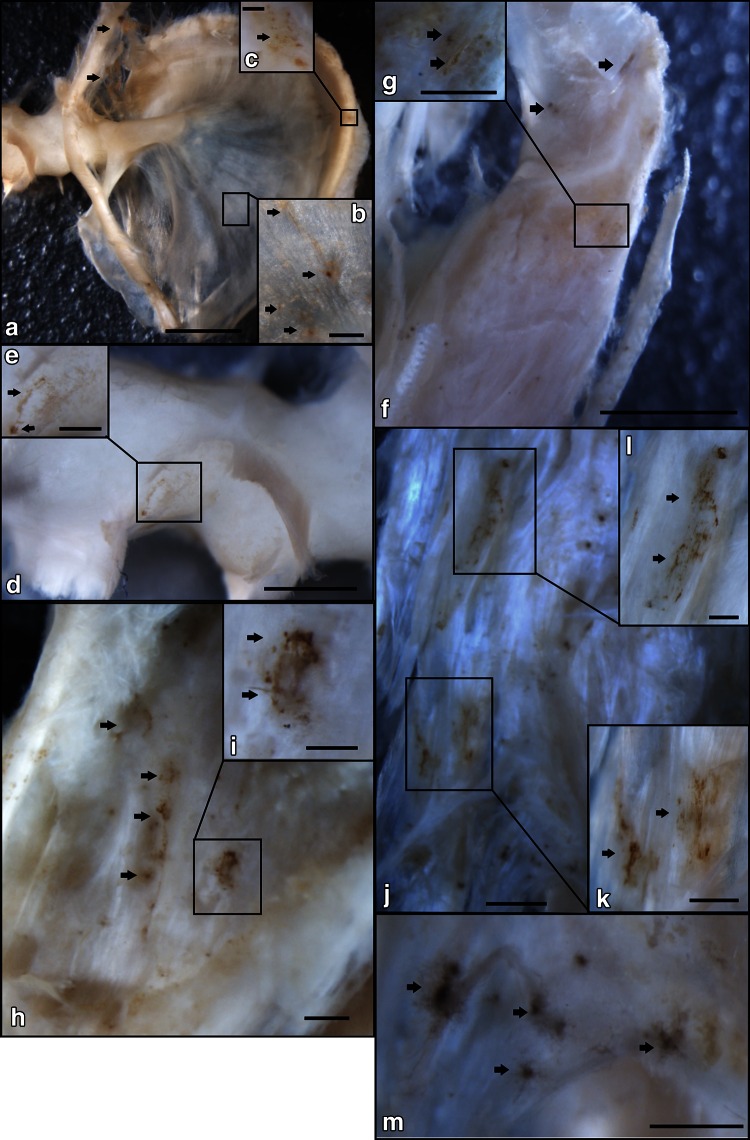
The distribution of Calcitonin gene-related peptide-immunoreactive nerve fibers (CGRP-IR NFs) in the inner surface region of the middle ear shown by immunohistochemical staining at the macroscopic level. **a** CGRP-IR NFs were located in the inner surface of the TM by anti-CGRP-IR NF immunohistochemical reactions (77-year-old female) (*bar* 2 mm). **b** Higher magnification of the square in **a**. CGRP-IR NFs (*arrows*) were sparsely located on the TM (*bar* 0.25 mm). **c** Higher magnification of the square in **a**. CGRP-IR NFs (*arrows*) were found in the fibrocartilaginous ring of the pars tensa of the TM (*bar* 0.1 mm). **d** Higher magnification of the neck of the malleus (see Fig. 1) (*bar* 1 mm); **e** Higher magnification of the square in **d**. A few CGRP-IR NFs (*arrows*) were observed (*bar* 0.5 mm). **f** The insertion site of the TTM on the malleus (see Fig. 1) (*bar* 2 mm); **g** Higher magnification of the square in **f**. CGRP-IR NFs (*arrows*) were observed (*bar* 0.5 mm). **h** The belly of the TTM (see Fig. 1). CGRP-IR NFs (*arrows*) were observed on the TTM (*bar* 0.5 mm). **i** Higher magnification of the *square* in **h**. Mesh-like structures of CGRP-IR NFs (*arrows*) were observed (*bar* 0.16 mm). **j** The connection site between the TTM and the TVPM (see Fig. 1) (*bar* 1 mm); **k** Higher magnification of the *square* in **j**. CGRP-IR NFs (*arrows*) were found in the vessel-like structures (*bar* 0.3 mm). **l** Higher magnification of the *square* in **j**. **m** The belly of the TVPM (see Fig. 1). Concentrated CGRP-IR NFs (*arrows*) were observed (*bar* 0.5 mm)

**Fig. 4:**
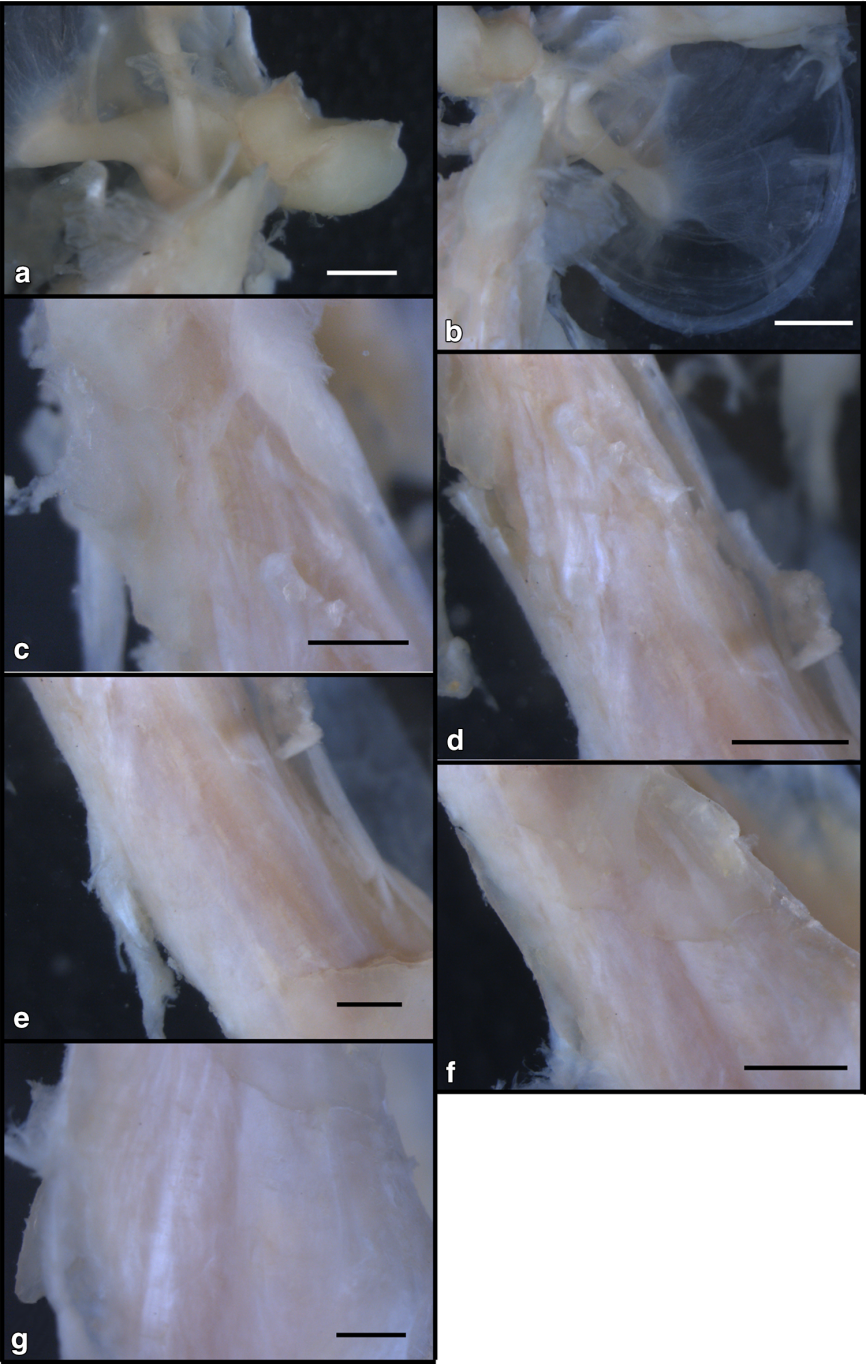
The negative control for SP and CGRP in the inner surface region of the middle ear is shown at the macroscopic level. **a** Inner surface of the tympanic membrane (*bar* 2 cm), **b** neck of the malleus, **c** insertion of the TTM at the malleus; **d**, belly of the TTM, **e** connection region between the TTM and TVPM, **f** belly of the TVPM (see Fig. 1) (46-year-old male) (*bar* 2 mm)

SP-IR NFs and CGRP-IR NFs were found around blood vessels located on the surface of the malleus bone, TM, TTM, and TVPM (Figs. 2, 3). SP-IR NFs and CGRP-IR NFs were scattered on the inner surface of the TM (Figs. 2a, b, 3a–c, 4a). In the neck of the malleus, SP-IR NFs were observed more frequently than CGRP-IR NFs (Figs. 2c, d, 3d–e, 4b). Numerous SP-IR NFs and CGRP-IR NFs formed a mesh-like structure located at the insertion of the TTM at the malleus (Figs. 2e, f, 3f, g, 4c), SP-IR and CGRP-IR NFs were scattered on the belly of the TTM (Figs. 2g, 3h, i, 4d). At the connective region between the TTM and the TVPM, SP-IR and CGRP-IR NFs formed a mesh-like structure scattered at the surface (Figs. 2h, i, 3j–l, 4e). SP-IR NFs and CGRP-IR NFs also formed a mesh-like structure that was clearly visible in the belly of the TVPM (Figs. 2j, k, 3m, 4f).

## Discussion

The TTM arises from the cartilaginous part of the auditory tube and the sphenopetrosal fissure of the sphenoid bone and inserts into the basal handle of the malleus. The TTM is a small muscle that receives arterial blood supply from the epitympanum branch of the middle meningeal artery and that is controlled by the TTM branch of the medial pterygoid nerve [15]. The TTM is connected to the tensor veli palatini muscle (TVPM) [15, 16]; however, it is unknown relationship between TTM and TVPM in the middle ear muscles. Histochemical observations have indicated that the TTM and TVPM function as a unit, and knowledge of their relationship is important for understanding the middle ear muscles [15].Therefore, examination of these sites of connection in the middle ear organ is important to understand the properties of the middle ear muscles. The TTM, TVPM and masticatory muscles have common innervation by the motor neurons of the trigeminal mandibular nerve. The TTM and TVPM function as a unit in movements [15]. The neurovascular insertion site in middle ear muscles may indicate important landmarks in the function of middle ear muscles. We examined the surface of six sites in the middle ear cavity using whole-mount immunohistochemical methods. The distribution of SP-IR and CGRP-IR NFs in the TTM, TVPM and related regions of the middle ear provides information regarding neurovascular levels associated with pain or inflammation. Therefore, we primarily analyzed the following six surface sites: the inner surface of the TM, neck of the malleus, insertion of the TTM at the malleus, belly of the TTM, connection region between the TTM and TVPM and belly of the TVPM.

### The inner surface of the TM

Vater–Pacinian corpuscles [17] and two types of cutaneous sensory nerve formations, the Meissner and Pacinian corpuscles, have been reported in the tympanic membrane [18]. In rat tympanic membranes, SP-IR and CGRP-IR NFs are located along blood vessels in the pars flaccid and in the fibrocartilaginous ring of the pars tensa [19]. In our study, SP-IR and CGRP-IR NFs were also found on the inner surface of the pars flaccid and the ring of the pars tensa of the human tympanic membrane. However, we did not find the fine structure of Vater–Pacinian corpuscles or Meissner and Pacinian corpuscles of cutaneous sensory nerves in the TM. SP-IR and CGRP-IR NFs were found only in certain regions of the inner surface of the human TM. These SP-IR and CGRP-IR NFs may act to control sensory terminals in the human TM.

### The neck of the malleus

Our results indicated that SP-IR and CGRP-IR NFs are concentrated at the surface of the neck of the malleus near the TM forming anterior and posterior pouches in humans (Prussak’s pouch) [20]. Prussak’s pouch is connected to the layers of the TM. The inner layer terminally attaches to the spina capitis mallei, which is situated on the transition from the neck to the head of the malleus [21]. However, in our study, the recess of the tympanic membrane was not clearly defined in the neck of the malleus, SP-IR and CGRP-IR NFs were detected in the connective tissue near the neck of the malleus, and SP-IR NFs were more frequently detected than CGRP-IR NFs in the region near the neck of the malleus of the cadavers. Autonomic nerve control of vasoconstriction may be located in the neck of malleus near the recess of the tympanic membrane.

### The surface region of the TTM and TVPM

In rat skeletal muscle, SP-IR and CGRP-IR NFs are more often found along blood vessels in the superficial muscle layers than in deep layers [22]. The different distribution and insertion of SP-IR and CGRP-IR NFs may be related to functional properties associated with blood supply for the muscle. We observed different localization of the SP-IR NFs and CGRP-IR NFs in our study of the human TTM and TVP. SP-IR and CGRP-IR NFs were mainly located at the insertion and the belly of the TTM, and connective region between the TTM and the TVPM, and the belly of the TVPM.

### Physiopathological hypotheses in pain and inflammation of TM and TVPM

These sites are sensitive areas of the TTM and TVPM in the middle ear. SP and CGRP are considered to be important mediators of neurogenic inflammation [23]. CGRP causes vasodilation and plasma extravasation, and simultaneous neurogenic release of CGRP in skeletal muscle may induce myofacial pain [24]. The malleus and the TM are also controlled individually or in combination by the TTM. The tendon of the long head of the biceps brachii is innervated by a network of sensory neurons that might indicate shoulder pain through the distribution of SP-IR and CGRP-IR NFs [25]. Therefore, the distribution of neural factors, such as SP-IR and CGRP-IR NFs, in these sites, may also reflect pain in the TTM and TVPM of the human middle ear. Our immunohistochemical observations indicated that SP-IR and CGRP-IR NFs are located in the insertion of the TTM at the malleus and in the connective region between the TTM and the TVPM. These areas induce pain or inflammation resulting from movements of these muscles.

Our results indicate that SP-IR NFs and CGRP-IR NFs are located in the connective areas between the two muscles. The existences of SP-IR NFs and CGRP-IR NFs may reflect muscle properties involved in pain or inflammation of the middle ear.
